# A highly sensitive famous face recognition paradigm for prosopagnosia screening

**DOI:** 10.3758/s13428-026-03033-w

**Published:** 2026-05-05

**Authors:** Sarah Bate, Emma Portch, Olivia Dark, Rachel Bennetts

**Affiliations:** 1 Department of Psychology, Faculty of Science and Technology, Bournemouth University, BH12 5BB Poole, UK; 2 Department of Psychology, Brunel University London, London, UK

**Keywords:** Face recognition, Face perception, Prosopagnosia, Semantics, Identification

## Abstract

Famous face recognition tasks have traditionally been used to diagnose prosopagnosia, offering striking examples of the inability to recognise highly familiar faces. Yet, their popularity has dwindled with the development of standardised unfamiliar face recognition tasks that are less cumbersome to administer and can readily be implemented online. Here, we argue that there is a danger of omitting measures of familiar face recognition from prosopagnosia screening: not only may this challenge the very definition of the condition, but, with some adjustments, famous face recognition tasks can continue to offer highly sensitive measures of everyday face recognition ability. Thus, we developed and evaluated an online, automated famous face recognition paradigm that can readily be implemented into large-scale screening programmes. This task improves on previous designs by (a) eliminating extrinsic cues to identity by including distractor as well as familiar faces, (b) supporting the use of unseen rather than “iconic” images of celebrities, and (c) offering a method for automated scoring. Multiple versions of the task were found to have high sensitivity in the detection of developmental prosopagnosia. When required, sub-scores collected from the same paradigm can be used to assess performance at different stages of recognition and identification, helping to probe more precise loci of impairment. The latter is important to guide the diagnosis of more complex cases and, potentially, their remediation.

Prosopagnosia, a relatively selective deficit in facial identity recognition, is typically diagnosed using multiple objective tests that assess face recognition ability (see Bate & Tree, 2017; Nørkær et al., 2024). While these tasks are sometimes supplemented with subjective self-report instruments (e.g., the 20-item prosopagnosia index: Shah et al., 2015; the prosopagnosia symptom checklist: PSC, Murray et al., 2018), it is generally agreed that impaired performance on at least two objective face recognition tasks is required for prosopagnosia diagnosis (DeGutis et al., 2023; Tree & Jones, 2025).

Traditionally, famous face recognition tests were the dominant objective tests used to diagnose face recognition difficulties in both acquired and developmental cases of prosopagnosia (Barton, 2008; Behrmann et al., 2005; De Renzi et al., 1991; Duchaine et al., 2007; Gauthier et al., 1999; Le Grand et al., 2006; Young et al., 1993), as well as other conditions that share this symptom (De Winter et al., 2016; Gefen et al., 2013; Rizzo et al., 2002; Valentine et al., 2006). Typically, famous face recognition tasks display a series of individually presented images of celebrities for an unlimited duration, and participants are required to respond with the person’s name or some uniquely identifying biographical fact. Such tasks are generally preferred to those involving the recognition of personally familiar faces, which require a bespoke set of images to be gathered for each participant, and incur difficulties in recruiting appropriately matched control participants who have had adequate exposure to the same target faces (e.g., Bate et al., 2015; Schmalzl et al., 2008). In contrast, famous face recognition tasks can be used across participants from a given country, with their data compared to a common set of control norms, and scrutinised to locate specific sources of error within the recognition process that can guide diagnosis and intervention. For instance, there are reports of individuals who are unable to make basic familiarity judgements about faces, indicating impairment at the early stages of recognition. In contrast, other reports suggest intact familiarity processing but difficulties recalling biographical information associated with that identity, or even the person’s name (e.g., Barton et al., 2001; Bate et al., 2015; Brunsdon et al., 2006; Young et al., 1993). Such neuropsychological case studies and the dissociations between them have informed theoretical frameworks of face recognition, where basic familiarity judgements are thought to occur at an early stage and can be separated from both the latter phase of semantic recall and the final step of accessing a person’s name (Bruce & Young, 1986). Pertinently, these stages of recognition can be tapped by different task demands in a famous face recognition task (i.e., by binary familiarity judgements, the recall of semantic or biographical information about a person, or by naming the face).

Despite these advantages, famous face recognition tests have reduced in popularity over the last 20 years and have often been replaced by widely available standardised assessments of unfamiliar face recognition that are used as the primary or sole means of diagnosis (but for advocation of continued use of familiar face recognition measures see Barton & Corrow, 2016; Bate et al., 2019; Murray et al., 2018; Shah et al., 2015). For instance, the dominant Cambridge Face Memory Test (CFMT; Duchaine & Nakayama, 2006) presents six unknown faces for encoding, and then probes recognition of those faces over 72 trials. The task takes approximately 12 min to complete, and has demonstrated excellent psychometric properties (Bowles et al., 2009; Murray & Bate, 2020; Wilmer et al., 2012). Clearly, there are multiple advantages of using tests such as the CFMT in prosopagnosia screening. Given the realisation that many people experience a developmental form of the condition (developmental prosopagnosia, DP; Bennetts et al., 2017; Bowles et al., 2009), there is now a need to screen large numbers of people from all over the world. The CFMT is appropriate for such purposes: alongside its fairly short duration, it can easily be deployed and automatically scored online, and does not require bespoke versions for different demographical groups (but see Bennetts et al., 2017; Croydon et al., 2014; Dalrymple et al., 2014; Kho et al., 2024; McKone et al., 2017, for alternative age and ethnicity versions of the task). In contrast, famous face recognition tasks not only need to be tailored to certain nations and age groups, but those that seek identification (either by naming or the provision of unique biographical information) rely on “free-type” responses, impeding automated scoring by the possibility of numerous correct answers and spelling errors. These paradigms require labour-intensive scoring by the experimenter (Bate et al., 2019), or rely on the participants themselves to honestly verify their own performance (cf. Wilmer et al., 2012; DeGutis et al., 2023).

In addition, most researchers (e.g., Bate et al., 2019; Bennetts et al., 2015; Duchaine et al., 2007; Mishra et al., 2019; Palermo et al., 2011; Pozo et al., 2022) agree that a further verification stage is required: participants need to be asked whether they *should* have recognised that face if they failed to do so (i.e., when presented with the celebrity’s name, the participant is required to indicate that they are sufficiently familiar with this individual; otherwise face recognition failures may be attributed to a simple lack of exposure to that person). Indeed, it is very unlikely that any one participant will have high familiarity with all celebrities in a famous face task (e.g., some may not watch movies or may not be interested in politics). In such scenarios, scores are adjusted by removing the celebrities that are unknown to participants by name, adjusting the proportion of correct responses accordingly. This correction imposes further demands on the scoring process (e.g., Bate et al., 2019; Palermo et al., 2011; cf. Wilmer et al., 2012).

Other advantages of highly controlled unfamiliar face recognition tasks (e.g., the CFMT) involve the images themselves: multiple pictures of each individual tend to be carefully captured, controlled and standardised by the authors. In direct contrast, images of celebrities are constrained by their availability. Historically, fewer images of celebrities were available and those that did exist were often of poor quality. This would force the use of “iconic” images, which were often presented in media reports and had likely been previously seen by participants. Participants might therefore be able to use pictorial image cues to identification (Carbon, 2008), and have often anecdotally reported the successful identification of a celebrity because they recognised “that particular picture”, or could even identify the movie or report that it had been extracted from (e.g., Bennetts et al., 2015). Fortunately, in today’s media and celebrity culture, numerous high-quality images of famous people are freely available online. Yet, there is still a need to avoid the use of the first images that result from Internet searches, which, depending on the search algorithm, are often taken from celebrities’ profile pages or have recently been featured in media reports.

Use of extraneous cues for identification is exacerbated by the fact that most famous face recognition tasks only present celebrity faces for identification (e.g., Bate et al., 2019; Duchaine et al., 2007; Wilmer et al., 2012): when no distractors are included, participants are aware that they *should* recognise every face in the set, cueing them to guess the identity despite not recognising the face at all. Some researchers attempt to overcome this issue by heavily cropping facial images around the internal features (i.e., excluding the hairline and sometimes even the jawline; Mishra et al., 2019; Pozo et al., 2022), but such adjustments have also been criticised for moving the task away from the ecological validity of everyday face recognition experiences (Burton, 2013).

Together, these difficulties underpin the increasing trend for researchers to diagnose prosopagnosia using performance on the CFMT, often supplemented by other unfamiliar face-processing tasks (e.g., the Face One in Ten task; Duchaine & Nakayama, 2005). Despite the clear efficiency of this approach versus the use of more laboursome famous face paradigms, there are still some clear advantages of the latter. Indeed, much research indicates a quantitative, if not a qualitative, difference in familiar versus unfamiliar face recognition ability (Burton, 2013; Megreya & Burton, 2006, 2007; Natu & O’Toole, 2011). For instance, while familiar face recognition has long been thought to rely on holistic or configural processing, it has been argued that unfamiliar face recognition does not (Megreya & Burton, 2006). Further, while most people excel at familiar face recognition, unfamiliar face recognition is much more difficult and elicits widespread individual differences in the typical population (Bindemann et al., 2012; Bruce et al., 1999; Hancock et al., 2000). These observations not only raise the possibility of independent processing streams that may be differentially affected, but the large standard deviations in the norming data of unfamiliar face recognition tasks also have important implications for prosopagnosia screening. Indeed, many suspected DPs achieve borderline scores that overlap with the distribution of scores in the typical population (e.g., Barton et al., 2019; Biotti & Cook, 2016). While this is typically overcome by the principle of administering multiple tests for diagnosis, it nevertheless differs from the distribution of performance that is typically observed on famous face recognition tasks: because most typical people excel at familiar face recognition, control norms tend to be very high with smaller standard deviations (Bate et al., 2019; Duchaine et al., 2007). This makes atypical performance particularly striking, as it will often fall multiple SDs away from the control mean. These differences in sensitivity imply that famous face tasks may be particularly useful in diagnostic practice: even if some flaws remain with methodological design, these could arguably be offset by a considerable improvement in sensitivity. Granted, the calibration of these tasks will not be suitable for the assessment of individual variation within the typical population, but they will offer a more definitive means of delineating impaired from typical performance in individuals with suspected face recognition difficulties.

There are further theoretical and philosophical implications of the above discussion. Indeed, one could question whether diagnostic practices have strayed too far from the original hallmark symptom of prosopagnosia—a profound deficit in *familiar* face recognition that was often positioned and tested as an amnestic rather than perceptual condition. Indeed, if we return to the pioneering prosopagnosia literature of the 1980s and beyond, neuropsychological case reports pointed to the striking loss of *familiar* face recognition skills following brain injury (e.g., Bruyer et al., 1983; De Renzi, 1986; Hecaen & Angerlergues, 1962). For instance, case descriptions often emphasise the difficulty that individuals with acquired prosopagnosia experience when recognising familiar people (e.g., famous faces, family), and even their own face (e.g., EM: Bate et al., 2015; HJA: Boutsen, 2002; PS: Rossion, 2003). Likewise, people with suspected developmental prosopagnosia self-refer for screening because they experience regular and striking failures of familiar face recognition that others do not (Murray & Bate, 2019). In fact, unfamiliar face recognition is something that is rarely considered in everyday life—we do not often receive feedback on our failures to recognise faces that we have only briefly seen before, and such instances are actually not uncommon to most people, irrespective of their face recognition ability (Young et al., 1985). Although unfamiliar face recognition tasks still place demands on memory, it is possible that our increased reliance on unfamiliar face recognition tasks at screening is inadvertently shifting the definition of prosopagnosia, diagnosing only individuals with deficits in earlier stages of the recognition process. For these reasons it is premature to eliminate familiar face recognition tasks from diagnostic practice, and we argue that they should be retained as a practically and theoretically informative aspect of prosopagnosia screening.

Thus, we sought to develop a novel famous face recognition paradigm that addresses the shortcomings of existing tasks, by (a) using less frequently seen rather than iconic images of celebrities, (b) including distractor trials (i.e., non-famous faces) to prevent cueing of identity, and (c) developing measures that could be automatically scored. Specifically, we aimed to evaluate whether our design could offer a highly sensitive means of prosopagnosia screening that could be readily implemented into large-scale online screening programmes. In an initial study, we evaluated the implementation of these design characteristics in a new famous face recognition paradigm using individuals that we already knew to have DP. This study also replicated the task across four versions that use different target celebrities to evaluate whether the basic paradigm could be adopted for use with different image sets as required (e.g., in different countries where alternative celebrities will be more familiar to participants).

Having demonstrated the use of automated scoring and the generalisability of the paradigm across different sets of images, Study 2 assessed its psychometric properties in a much larger set of participants. Further, we continued to assess the paradigm’s generalisability by adopting a novel set of target stimuli. This much larger dataset was also used to present norming data for comparison to DPs. In Study 3, we applied these norms to a group of participants who reported to our lab in the belief that they had DP. By administering the paradigm to individuals who did not yet know whether they had a confirmed DP diagnosis, we were able to assess the sensitivity of the task for detecting face recognition deficits without any bias from previous experiences with face recognition tasks or knowledge about their performance on those assessments.

## Study 1

An initial study assessed the viability and generalisability of a novel famous face recognition paradigm as a means to detect DP, improving on existing designs by avoiding the use of iconic images and by including an equal number of distractor trials. Our specific aim was to use this paradigm to determine whether automated scoring measures can offer comparable diagnostic sensitivity to those that require manual (and therefore labour-intensive) scoring. We included two measures in our design that could be automatically scored: a binary familiarity judgement for each face and a forced-choice semantic classification (i.e., choosing one of 13 occupational categories that best describe why the target is famous, such as author, actor, or entrepreneur). We compared the sensitivity of these two measures to a third measure that required free-type entry of individuating information about the person (e.g., their name or a specific movie that they appeared in), and therefore manual scoring by the experimenters. Finally, we applied this paradigm to four different versions of the task in order to assess its generalisability across different stimulus sets.

### Method

#### Participants

Because the scoring of this study was labour-intensive (i.e., manual scoring was required for one measure per familiar trial across four blocks), we set our target sample size at 25 participants with a pre-existing diagnosis of DP (see Supplementary Material for diagnostic screening procedures and scores: https://osf.io/wa56h). These individuals had taken part in our previous work (Bate et al., 2019, 2025) and were aged 26–59 years (*M* = 45.2, *SD* = 10.2; 17 female). Fifty control participants aged 24–64 years (*M* = 44.6, *SD* = 10.5; 33 female) were also recruited via local advertising on social media. All participants had lived in the UK for their entire life, and no participant reported any history of neurological, psychiatric or visual conditions. Participants provided informed consent to take part in the study in exchange for a financial incentive, and ethical approval was granted by the institutional ethics committee.

#### Materials

The top 100 identities (35 female—familiarity was favoured above gender balance; see Supplementary Material: https://osf.io/wa56h) were selected from a government survey of the individuals most well-known to adult citizens in the UK^1^. To avoid the use of iconic images, each celebrity’s name was entered into a Google image search, and the 100th image that was returned was selected for use in the study. If the image in the 100th position was not deemed suitable (i.e., because the face was obscured, the individual was not facing the camera, image quality was low, or it replicated a higher-ranked image), the proceeding most suitable image was selected.

To avoid cues that all faces were celebrities and to assess the familiarity stage of recognition in a meaningful way, the paradigm improved on previous tasks by including an equal number of distractor images that were each matched to a celebrity identity. To select distractors that could plausibly be perceived to work in the entertainment industry (but who were not famous), we selected stimuli from the webpage of an actor “extras” agency. One distractor was matched to each celebrity according to gender, age, and perceived attractiveness. The initial matches were made by one member of the research team and were then checked for agreement by a second researcher.

We made the decision to crop all images below the chin, without excluding any external features (i.e., the full head, including the hair, was visible). While other famous face tasks have cropped faces much more tightly around the inner facial features, we wanted to include the full head for reasons of ecological validity (to reflect everyday face recognition when this visual information is nearly always available; Burton, 2013) and also to assess the task with minimum image preparation. Nevertheless, we ensured that no cues to identity could be inferred by the background of any of the pictures, but we did not standardise the background. All images were adjusted to 400 pixels in height, and the width was permitted to vary to prevent image distortion.

This resulted in a pool of 200 images: one of each of 100 celebrities and one of each of 100 matched distractors. In order to allow screen breaks and to assess the generalisability of the design, the 200 images were then split into four blocks of 50, each containing 25 celebrities and the relevant 25 distractors.

#### Procedure

The task was completed online and remotely in participants’ homes, using the Qualtrics testing platform. The order of the four blocks was randomised for each participant, and all trials were randomised within each block.

**Fig. 1:**
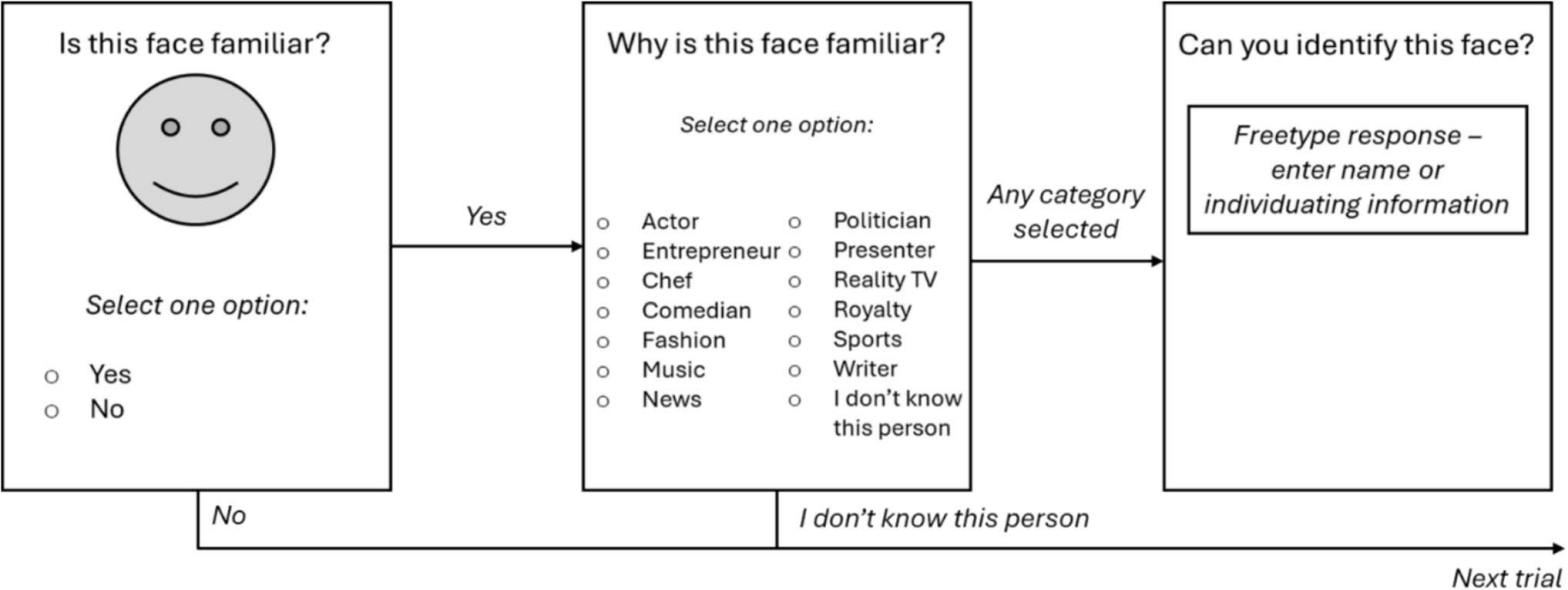
A schematic summary of each trial. Faces were initially presented for a binary familiarity judgement. If familiarity was indicated, participants were asked to select the semantic category that best fit that person, with an option to declare that they did not know the person. If a semantic category was selected, they were asked to free-type the person’s name or some individuating information about that person. If familiarity was not indicated, or the participant declared they did not know the semantic category for that person, the task immediately proceeded to the next trial. This procedure was followed for both famous and distractor trials. Note that the semantic categories presented in the figure are abbreviated

In each trial, the target face was displayed at the top of the screen. It remained on the screen for an unlimited time until participants made a yes/no response to the question “Is this face familiar?” If they responded “no”, the next trial appeared. If they responded “yes”, the face disappeared and they were asked to respond to some further questions that permitted examination of performance at later stages of recognition, with no time limit imposed (see Fig. 1).

The first of these questions was designed to introduce a means of automated scoring for the latter stages of recognition. Previous paradigms have relied on free-type entry of names or biographical information, which prohibits automated scoring due to the possibilities of spelling errors and numerous correct responses. Here, participants were asked “Why is this face familiar?”, and were asked to select one of the following 13 categories (with chance being 7.69%): actor or director; business person or entrepreneur; chef or cookery personality; comedian; fashion designer or model; music artist; newsreader, journalist or current affairs (any medium); politician; presenter or broadcaster (entertainment: any medium or topic); reality television or social media personality (contestant, judge, or star); royalty (family member with or without title); sports star or personality; writer. There was also an option to state “I don’t know who this person is”. These categories were selected to provide an exhaustive list of the primary occupations of all celebrities in the stimulus set, as agreed by two experimenters. Participants were instructed to categorise celebrities based on their most recent and/or most typical activities.

If participants selected the “I don’t know who this person is” option, they immediately proceeded to the next trial. For all other responses, participants were then asked to “enter one piece of uniquely identifying information about this person, or their name (e.g., a movie character that they have played, a song they have performed, a programme they have presented or a public role they have filled).” Collection of this information allowed us to examine whether the data collected in the preceding semantic categorisation phase provided an equally accurate and sensitive measure of recognition. All the steps outlined above (see Fig. 1) were also presented when distractor stimuli were incorrectly judged to be familiar.

Finally, after all blocks were completed, participants completed a questionnaire where they viewed the names of the 100 celebrities that they had seen in the study. They were instructed: “To ensure the integrity of our data, we need to establish whether any recognition errors result from a genuine failure to recognise that person, or simply because you have no or little knowledge of that person per se (e.g., if you are not interested in sports or movies, you would be unlikely to recognise certain sports stars or actors even if you had excellent face recognition skills).” For each name, they were asked to rate their familiarity with that person on a Likert scale ranging from 1 (not at all familiar) to 5 (very familiar). The exposure questionnaire was always completed last so that participants were not cued by the names of the celebrities before seeing their faces. Participants were encouraged to take breaks between all blocks, but not within a single block.

#### Statistical analyses

Response time was not analysed given the multiple pieces of information that needed to be entered for each face. Celebrity trials were each scored on three parameters: those correctly identified as familiar, those correctly categorised by semantic occupation, and those for which correct individuating information was provided (by entering the person’s name or some uniquely identifying biographical detail). Familiarity and categorisation responses were automatically scored, whereas naming and/or provision of unique biographical information required manual scoring. Two experimenters had previously agreed upon the “correct” semantic category/categories for each celebrity (note that, in many cases, the most recent or most typical activities for celebrities varied, and all applicable categories were therefore considered correct). However, we accepted additional categories as correct answers when they had been selected by more than 15% of the control sample (the number of participants selecting additional categories varied for each celebrity, ranging from 2 to 17 individuals) and this corresponded with an accurate reflection of the celebrity’s portfolio. This cut-off also coincided with being numerically above the average frequency with which any additional categories were selected by control participants. The free-type naming/identifying information responses were initially scored by one experimenter, with any ambiguous responses checked for agreement by a second experimenter.

Correct responses on all measures were then separately summed per participant for each block. The overall proportion correct for each of the famous face measures was adjusted for each participant by removal of the celebrities identified as unfamiliar (ratings of 1 or 2 on the Likert scale) by name in the end-of-task questionnaire. While this control measure does not equate exposure to each celebrity across participants, it does ensure that each individual participant has had at least some visual experience with each target face.

Correct rejections were also summed for the distractor stimuli. These data were combined with the familiarity judgement responses for celebrity trials (hits) to calculate signal detection theory measures of sensitivity (*A*) and response bias (*b*) (Zhang & Mueller, 2005) (henceforth referred to as “familiarity sensitivity” and “familiarity bias”, respectively). *A* and *b* were selected due to the non-normal distribution (negative skew) of hits and false alarms. Values of *A* range from 0 to 1, with 0 indicating chance-level performance and values closer to 1 indicating better performance. Values of *b* around 1 suggest a neutral response bias; those lower than 1 indicate a liberal response bias—that is, a tendency to respond “familiar”; values higher than 1 indicate a conservative response bias—that is, a tendency to respond “unfamiliar”.

We also used signal detection measures (Macmillan & Creelman, 2005) to calculate the sensitivity (*d′*) of each measure (familiarity judgement, semantic categorisation, free-typed identification information) to categorise cases of DP (henceforth referred to as “categorisation sensitivity”), and the level of bias in categorisation (*c*). The number of participants with DP who were correctly categorised as impaired (> 1.7 SDs below the control mean^2^) were considered hits; the number of control participants who were incorrectly categorised as impaired were considered false alarms. Extreme values were replaced as per recommendations in Stanislaw and Todorov (1999). For these data, a *d′* of 0 would indicate chance-level categorisation, and a *d′* of 4.38 would indicate perfect categorisation performance. Finally, we carried out receiver operating characteristic (ROC) analyses to examine and compare the accuracy of the tests and measures at different cut-offs (see Supplementary Materials for full ROC results: https://osf.io/wa56h).

### Results

#### Data overview

Initial analyses used control data to examine the suitability of the target celebrities for the breadth of the age group that was tested. No significant correlations were observed between participant age and the number of celebrities removed from the analysis due to low familiarity in any of the four blocks (all *p*s > .202). To examine whether the number of famous faces that were unknown to participants was consistent across blocks, we performed a 4 (Block: A, B, C, D) × 2 (Group: controls, DPs) mixed-measures analysis of variance (ANOVA). This resulted in a significant main effect of Block, *F*(3, 219) = 7.206, *p = *.001, η_ρ_^2^ = .090, and follow-up analyses indicated that more celebrities were removed from Block A (*M* = 3.61, *SE* = 0.51) than from the other three blocks (*M*s = 2.51, 2.83, 2.84, respectively; *SEs* = 0.39, 0.41, 0.48; *p*s < .007, η_ρ_^2^s > .095), with no differences between the latter three blocks (*p*s > .105). The main effect of Group and the interaction between Block and Group were both non-significant (*p*s > .140). Thus, while slightly more faces were unknown in Block A, more than 88% of famous trials were on average retained in each block, with no differences between controls and DPs in their previous exposure to the targets.

Next, we examined the convergent validity of the four blocks of the task by correlating DP scores on identification of the target celebrities with the same measure of their performance on our existing famous face task used at screening (see Supplementary Material: https://osf.io/wa56h; note that we only hold identification-level data for this task and therefore only compared data to the corresponding measure). This produced a moderate effect size in all four blocks (*N* = 25; *r*s* = *.616, .568, .402, .700; *p*s = .001, .003, .046, .001). Inter-item reliability was also high: both controls (all *r*s > .386, all *p*s < .006) and DPs (all *r*s > .589, all *p*s < .002) demonstrated high correlations in their celebrity face recognition performance across all three recognition measures on the four blocks.

#### Familiarity judgements of famous versus novel faces

**Fig. 2:**
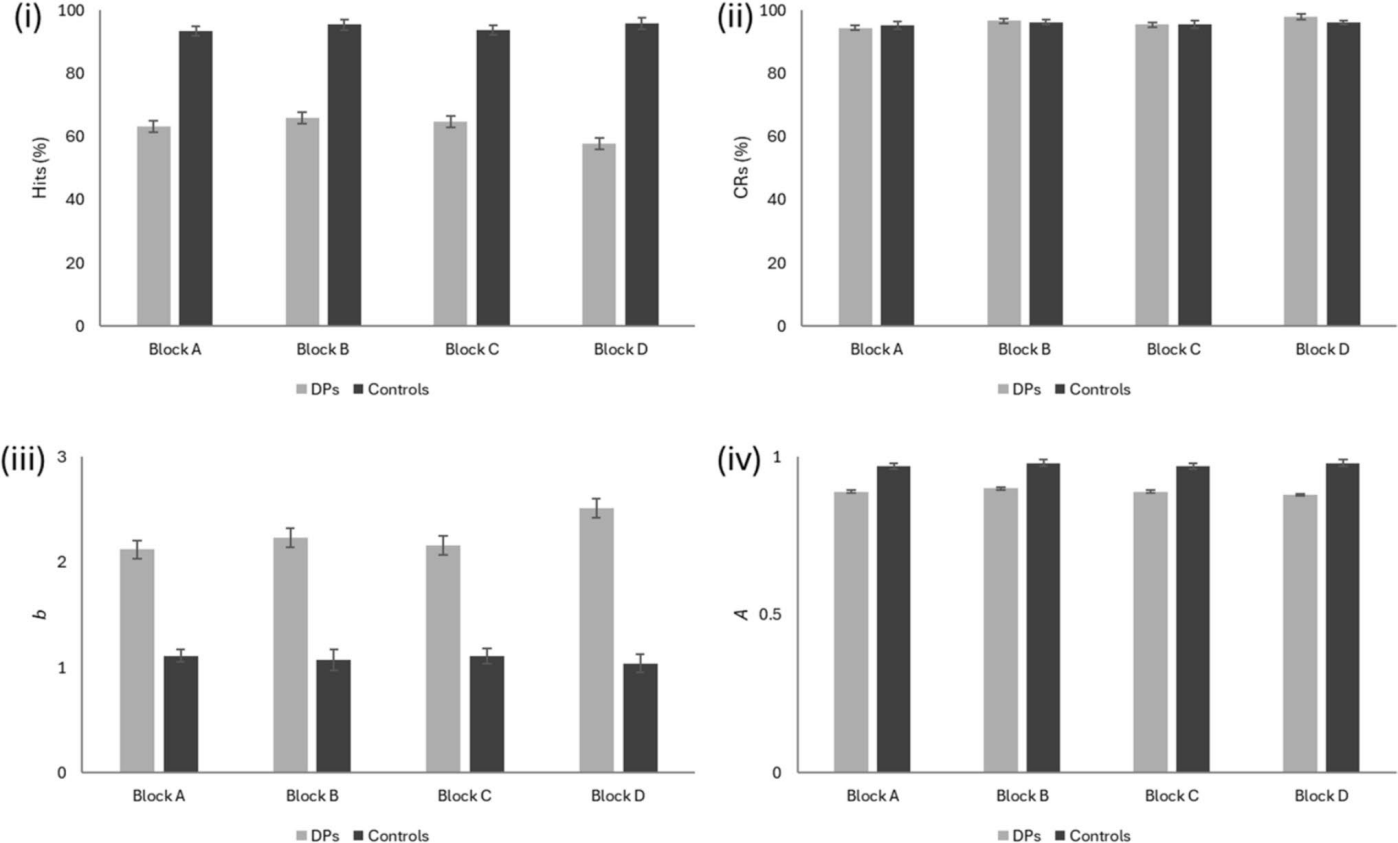
Performance of DPs and controls across the four blocks according to (i) correct familiarity responses for celebrity faces (hits), (ii) correct unfamiliar responses for distractor faces (correct rejections), (iii) response bias (*b*), and (iv) sensitivity (*A*)

Having looked at the psychometric properties of each measure of the task, we then examined differences in performance on each measure in each block across DPs and controls. First, we examined hits (i.e., correct familiarity responses for famous faces). A 4 (Block) × 2 (Group) mixed-effects ANOVA yielded a significant interaction, *F*(3, 219) = 6.683, *p = *.001, η_ρ_^2^ = .084 (see Fig. 2). Follow-up contrasts corrected for multiple comparisons revealed that DPs performed better in Block B (*M* = 65.92%, *SE* = 2.32) than in Block D (*M* = 57.74%, *SE* = 2.50; *p* = .004, *d* = .425), and controls performed better in Block D (*M* = 95.74%, *SE* = 1.77) than in both Blocks A (*M* = 93.35%, *SE* = 1.59, *p = *.006, *d* = .403) and C (*M* = 93.65%, *SE* = 1.50, *p = *.010, *d* = .377). This superseded a significant main effect of Block, *F*(3, 219) = 4.025, *p = *.008, η_ρ_^2^ = .052, whereby follow-up analyses corrected for multiple comparisons indicated that performance in Block B (*M* = 80.69%, *SE* = 1.42) was better than in Block D (*M* = 76.74%, *SE* = 1.53; *p = *.001, η_ρ_^2^ = .158). Further, a main effect of Group indicated that controls (*M* = 94.55%, *SE* = 1.41) outperformed DPs (*M* = 63.27%, *SE* = 2.25), *F*(1, 73) = 168.637, *p = *.001, η_ρ_^2^ = .698. Despite these differences in the calibration of blocks, the categorisation sensitivity of all four blocks using hits was very high (see Table 1).

**Table 1:** Famous face recognition performance on each of the four blocks for each of the three recognition measures. The 1.7-SD cut-offs are provided for each block and measure, together with the sensitivity (*d′*) for classifying each individual as a DP or control

	Block	Control mean	Control *SD*	1.7-SD cut-off	1.7-SD DP hits	1.7-SD Control hits	1.7-SD *d′*
Sensitivity (*A*)	A	0.97	0.03	0.92	18/24	47/50	2.23
	B	0.98	0.02	0.95	22/24	42/50	2.38
	C	0.97	0.04	0.90	15/24	45/50	1.60
	D	0.98	0.03	0.93	21/24	47/50	2.71
Familiarity	A	93.35	6.86	81.69	21/25	46/50	2.40
	B	95.46	5.89	85.45	24/25	46/50	3.16
	C	93.64	8.97	78.39	22/25	44/50	2.35
	D	95.74	6.24	85.13	24/25	45/50	3.03
Semantic	A	90.80	8.34	76.62	22/25	46/50	2.58
	B	92.60	7.63	79.63	21/25	47/50	2.55
	C	90.23	9.96	73.30	21/25	46/50	2.40
	D	94.17	7.61	81.23	24/25	48/50	3.50
Identification	A	90.48	8.71	75.67	23/25	46/50	2.81
	B	92.75	7.22	80.48	25/25	46/50	3.46
	C	88.91	11.26	69.77	20/25	47/50	2.40
	D	92.78	9.11	77.29	23/25	45/50	2.69

Analysis of correct rejections did not reveal a similar picture, suggesting that as a measure on its own it has little diagnostic value. A 4 (Block) × 2 (Group) mixed-effects ANOVA did not yield any significant interaction or main effect (*p*s > .217). Given that this could be a product of response bias in DPs (e.g., an increased likelihood of rejecting stimuli as unfamiliar), we repeated the same ANOVA using *b*^3^. Here, there was a significant interaction between Block and Group (see Fig. 1), *F*(3, 216) = 4.556, *p = *.007, η_ρ_^2^ = .060, whereby DPs responded more conservatively in Block D than in the other three blocks, *F*(1, 23) = 7.400, *p = *.012, η_ρ_^2^ = .243, but there were no differences in control performance (*p* > .05). This superseded the main effect of Group, whereby a more conservative response bias was observed in DPs (*M* = 2.24, *SE* = 0.09) than in controls (*M* = 1.08, *SE* = 0.07), *F*(1, 72) = 107.102, *p = *.001, η_ρ_^2^ = .598. Finally, the main effect of Block was not significant (*p* = .102).

Given the influence of bias, we then repeated the same ANOVA using the familiarity sensitivity measure *A*. This revealed a significant main effect of Group, whereby controls (*M* = 0.97, *SE* = .01) outperformed DPs (*M* = 0.89, *SE* = .01), *F*(1, 72) = 89.835, *p = *.001, η_ρ_^2^ = .555. There was no main effect of Block or an interaction with Group (*p*s > 0.188).

Across all blocks, the categorisation sensitivity *d′* was higher for hits than for familiarity sensitivity *A*. Given that the calculation of *A* can be complex (for example, different equations may be used depending on the values of hits and false alarms for each participant; see Zhang & Mueller, 2005), and there is no obvious benefit in terms of categorisation sensitivity, researchers may wish to focus solely on hits when analysing performance on famous face familiarity judgement tasks.

#### Performance on different familiar face recognition measures

The next analysis aimed to compare measures of the different stages of recognition, to assess whether familiarity or semantic-level judgements are the best indicators of DP, and whether an automated measure of semantics will perform comparably to one that needs to be manually scored. To address this, a 3 (Measure: familiarity hits, semantic categorisation, free-type identification) × 2 (Group: DP, control) mixed-measures ANOVA was performed for each block.

**Fig. 3:**
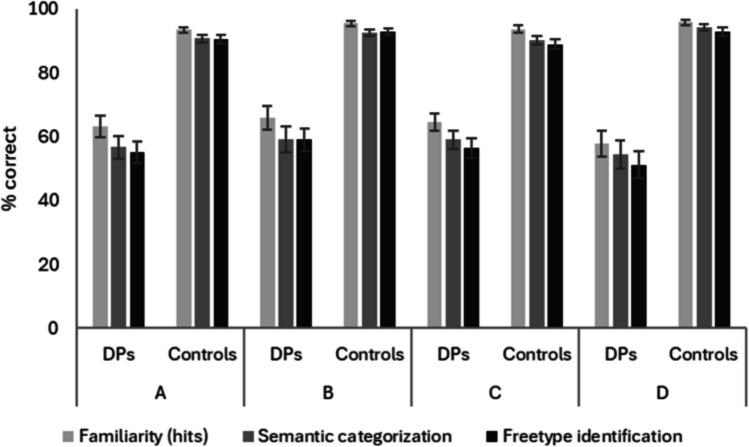
Performance (% correct) for DPs and controls on each of the Blocks A to D, for familiarity (hits), semantic categorisation, and the free-type entry of unique biographical information (either semantic information or names). While free-type entry was manually scored, familiarity and semantic categorisation were automated measures

The main effects of Group indicated that controls outperformed DPs in all four blocks (all *p*s < .001, all η_ρ_^2^ > .640). There were also significant main effects of Measure in all four blocks (all *p*s < .001, all η_ρ_^2^ > .205); follow-up analyses confirmed that performance on the familiarity measure was greater than on the semantic measure in all four analyses (all *p*s < .001, all η_ρ_^2^ > .215), and better on semantic categorisation than free-type identification in Blocks C and D (*p*s < .004, η_ρ_^2^ > .106; other *p*s > .106; see Fig. 3).

The ANOVAs also resulted in a significant interaction between Measure and Group for all blocks (*p*s < .047, η_ρ_^2^ > .043) other than Block C (*p* = .078). Follow-up analyses confirmed that controls outperformed DPs on all measures in all blocks (all *p*s < .001; all *d*s > 2.52). However, while both DPs and controls achieved higher scores on the familiarity compared to the semantic categorisation measure in all blocks (all *p*s < .010, all *d*s > .564; see Fig. 3), the difference between scores was greater in DPs than in controls in Blocks A and B (*p*s <.038, *d*s > .66) but not C and D (*p*s > .120). Further, there was a difference between the semantic categorisation and free-type scores in DPs (*p* = .010, *d* = .563) and controls (*p* = .008, *d* = .393) only in Block D.

The ANOVAs also resulted in a significant interaction between Measure and Group for all blocks (*p*s < .047, η_ρ_^2^ > .043) other than Block C (*p* = .078). Follow-up analyses confirmed that controls outperformed DPs on all measures in all blocks (all *p*s < .001; all *d*s > 2.52). However, while both DPs and controls achieved higher scores on the familiarity compared to the semantic categorisation measure in all blocks (all *p*s < .010, all *d*s > .564; see Fig. 3), the difference between scores was greater in DPs than in controls in Blocks A and B (*p*s <.038, ds > .66) but not C and D (*p*s > .120). Further, there was a difference between the semantic categorisation and free-type scores in DPs (*p* = .010, *d* = .563) and controls (*p* = .008, *d* = .393) only in Block D.

Finally, Table 1 indicates that categorisation sensitivity was high, and relatively similar, for all measures in all blocks. This conclusion is supported by ROC analyses: analyses of the area under the ROC curve (AUC) showed that all measures performed well (range 0.85–0.98) and were significantly better than chance at discriminating between DP and control participants (see Supplementary Material for further details: https://osf.io/wa56h). An ANOVA on categorisation sensitivity with measure as the independent variable (*A*, familiarity hits, semantic categorisation, identification) found no significant difference in categorisation sensitivity for the four measures, *F*(3, 9) = 2.99, *p* = .09. Once again, this is supported by the ROC analyses, which found minimal differences in the AUC for different measures. This suggests that there is little diagnostic advantage of using free-type identification information (which requires manual scoring) over the familiarity judgement and semantic category information (which can be automatically scored) when screening for DP.

## Discussion

Study 1 aimed to evaluate a new famous face recognition paradigm for prosopagnosia screening that overcomes the limitations of existing paradigms. Thus, we created a task that (a) prevented identification cueing by including distractor as well as famous faces (simultaneously allowing for an automated measure at the familiarity stage), (b) used lesser-seen rather than iconic images of celebrities, and (c) evaluated whether task sensitivity can be retained when introducing measures that allow for automated scoring, and across different blocks containing different celebrities and distractors. Findings indicated that the use of automated scoring measures offers a sensitive alternative to free-type identification responses that are laboursome to score.

The paradigm used here improved upon existing face recognition tasks in several ways, although it is acknowledged that we did not experimentally manipulate all factors to provide directly supporting evidence. First, rather than only displaying celebrities for identification, it included an equal number of distractor and celebrity faces and additionally measured the accuracy of familiarity judgements. Here, unsurprisingly, we noted a difference in response bias between DPs and controls, whereby DPs responded more conservatively and were more likely to indicate that faces were unfamiliar to them than controls. This is an important improvement on existing paradigms, where only celebrity stimuli tend to be shown. Because participants are aware that all faces should be familiar to them, they may take educated guesses on identity based on individual distinguishing features or image-based cues. Although we cannot statistically show that this is an improvement on designs that do not contain distractor stimuli, we are aware from our previous (extensive) use of such paradigms that DPs often comment that they know the face should be familiar to them, and they can therefore guess at the identity, often using pictorial cues to recognition (see Portch et al., 2023). In the current design, there is an equal chance that the face is a distractor, removing the prompt that every trial depicts a familiar identity. It is possible that the participants were guessing the semantic category of familiar faces based on judgements from pictorial information rather than person recognition itself. While we do not have data to directly assess this possibility (i.e., when participants indicated they were unfamiliar with a celebrity’s face, they were not prompted to provide a semantic category), it is of note that the semantic categorization measure correlated highly with the manual free-type measure that required the input of individuating information. Thus, we think it unlikely that non-identity recognition processes aided the semantic categorization component of the task.

Our use of lesser-seen images also helped to address this. Strikingly, sensitivity remained high even though we did not crop any faces of their external features. Many existing famous face DP screening tasks crop celebrity facial images, at least around the hairline, to prevent external cues to recognition (Mishra et al., 2019; Pozo et al., 2022). While this may seem particularly important for highly familiar faces, some authors have suggested that the removal of outer facial features essentially changes the task itself, imposing an unnatural paradigm that does not mirror face recognition in the real world (Burton, 2013). Although we did not experimentally manipulate this factor within the current study, the data presented here nevertheless provide an important demonstration that task sensitivity can be retained without image cropping, even in a highly familiar face recognition task. While the calibration of the task is likely only suitable for distinguishing impaired from typical participants, as opposed to identifying more subtle individual differences in performance, the sensitivity levels demonstrated by our paradigm are robust (maximum *d′* = 3.50). Thus, while we cannot exclude the possibility that some DPs were able to use pictorial cues for successful recognition in some trials, the robust sensitivity values across blocks suggest that compensatory strategies were not able to consistently support successful performance.

Indeed, categorisation sensitivity (*d′*) and AUC were relatively consistent and high across all versions. Thus, the basic paradigm generalised across different versions that used different target identities, despite some minor differences in calibration. Further, it is clear that each individual block had sufficient sensitivity for independent use in prosopagnosia screening, indicating that a single streamlined version of the task containing only one block of 50 trials (25 celebrities, 25 distractors) would be sufficient in practice. Categorisation sensitivity and AUC were also remarkably consistent across the different measures of performance, suggesting that all measures have the potential to be useful in screening for DP. However, other factors may influence the choice of measure to be used in research settings. While the signal detection measure *A* offered high categorisation sensitivity in most blocks, numerically it was slightly less sensitive than other, more straightforward measures of recognition (i.e., hits, semantic categorisation); further, it is complicated to calculate and understand for lay users. The free-type identification measure was no more sensitive than familiarity hits or semantic categorisation, and the automated scoring offered by either of these two measures could provide sensitive and efficient measures in large-scale screening. While the sensitivity and AUC did not differ between familiarity hits and semantic categorisation measures, researchers may take comfort from the additional engagement required for semantic categorisation and the reduced odds of correct scores resulting from “lucky guesses” (i.e., 50% chance of a correct guess at the familiarity level, compared to 7.69% at the semantic level). As such, it may be advisable to include both familiarity judgement and semantic categorisation in screening tasks. These data may also be of particular interest when examining specific hypotheses for particular cases (perhaps beyond the scope of DP), such as when familiarity judgements are suspected to be preserved yet identification impaired.

In sum, we concluded from this study that our new paradigm offers a sensitive means of detecting prosopagnosia that is consistently successful across automated scoring measures. Because this study required a large amount of labour-intensive manual scoring, we had set a target sample size that was sufficient to demonstrate the generalisability and sensitivity of the new paradigm, but was not large enough to thoroughly evaluate its other psychometric properties (e.g., reliability), and the range of performance in unimpaired perceivers. Our next step was therefore to assess these metrics in a much larger sample of participants, using a single block of trials.

## Study 2

Having demonstrated the sensitivity of automated scoring measures in the new famous face paradigm, our second study aimed to assess the wider psychometric properties of the paradigm in a much larger set of typical participants. This approach is commonly applied to the development of new psychometric tasks, where the range of performance and reliability of the task is explored in the typical population, together with the development of a set of norming data for comparison to those reporting with deficits (e.g., Murray et al., 2022).

Because we aimed to assess a large number of participants in this task, and we had demonstrated the viability of automated measures in Study 1, we only took the two automated measures forward into Study 2 (i.e., hits at the level of familiarity judgements and semantic categorisation accuracy). This permitted us to avoid labour-intensive scoring of free-type measures. Finally, we continued to assess the generalisability of the paradigm by using a new set of stimuli, but proceeded with a single block of trials given that all four blocks in Study 1 independently demonstrated appropriate sensitivity, advocating for a more streamlined approach.

### Method

#### Participants

Previous studies have reported moderate correlations between famous face tests and those tapping face memory, with effect sizes ranging from around 0.33 to 0.50 (e.g., Bate et al., 2019; McCaffery et al., 2018; Pozo et al., 2022). A sample size calculation with an effect size at the lower end of this range (*r* = .30) and power of 0.9 indicated that 110 participants would be sufficient to detect a significant effect (G*Power 3.1). However, sample size calculations for reliability indicated that 150 participants would be required to estimate reliability effectively (based on estimated reliability of 0.8, 26 items, and power of 0.9; Arifin, 2018).

We therefore aimed to exceed a sample size of 150, with our funding allowing a total of 174 typical participants to be recruited via the online participant database Prolific. Participants were aged 20–64 years, and all had lived in the UK their entire lives. No participant reported any history of neurological, psychiatric, visual, or developmental conditions. Eight participants were removed from the dataset due to unusually poor performance (more than 3 SDs from the mean) on the tasks, suggesting poor engagement. This resulted in a final dataset containing 166 individuals (98 female) aged between 20 and 64 years (*M* = 39.2, *SD* = 11.1).

#### Materials and procedure

The paradigm used in Study 2 was identical to Study 1, with the exception of one minor adjustment to the number of stimuli. In Study 1 we had used 25 celebrity images and 25 matched distractors per block: here, we created one new block that contained 26 identities. This slight adaptation allowed us to balance the gender of the target faces (i.e., 13 female and 13 male). Again, we selected the famous identities from the same government survey of the individuals most well-known to UK adults^1^. As Study 2 was completed in the year following Study 1, this resulted in a different set of target stimuli (see Supplementary Material: https://osf.io/wa56h), offering a further assessment of the generalisability of the paradigm to a novel set of stimuli. Celebrity and distractor images were selected and prepared in an identical manner to Study 1. This resulted in a pool of 52 images: 26 celebrities and 26 matched distractors. Images were presented in random order using the same platform, paradigm, and response options as described for each block of Study 1.

To assess convergent reliability, participants were also asked to complete the CFMT (Duchaine & Nakayama, 2006), and the Cambridge Bike Memory Test (CBMT: Barton et al., 2019) was also administered as a test of divergent reliability. The CFMT presents six unknown faces for encoding and then probes recognition of those faces over 72 trials. The CBMT uses the same format as the CFMT, but the stimuli are bicycles.

### Results

**Fig. 4:**
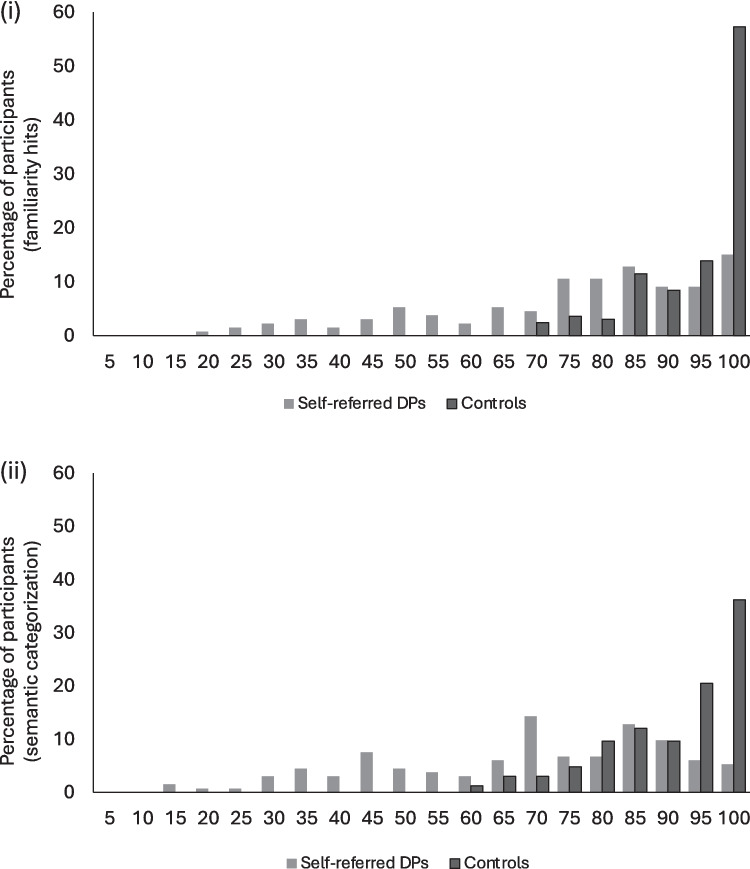
Distribution of performance in the new famous face test, for (i) familiarity judgements and (ii) semantic categorisation. Black bars show the percentage of typical participants (controls) performing in each accuracy range (Study 2). Grey bars show percentage of self-reported DP participants (self-referred DPs) performing in each accuracy range (Study 3)

First, we examined performance on the famous face test using the measures identified in Study 1—namely, familiarity hits and sematic categorisation. Performance in the overall sample ranged between 66.67% and 100% on the familiarity measure, and between 57.14% and 100% on the semantic measure (see Fig. 4). Table 2 presents descriptive data for the overall sample, and further norms as a function of participant age. However, there was no significant correlation between age and either the familiarity (ρ = .011, *p* = .891) or semantic (ρ = .062, *p* = .428) measure. Further, age did not correlate with any of the other tasks in the battery (*p*s > .097). There was a correlation between age and the number of celebrity names that the participant regarded as unfamiliar, resulting in removal of those corresponding recognition trials (ρ = −.237, *p = *.002). Follow-up group-based analyses indicated that the difference resided between those in their 20s (*M* = 5.76, *SE* = 0.79) and 30s (*M* = 3.38, *SE* = 0.60) (*p = *.019,* d = *.518, other *p*s > .227).

**Table 2:** Performance on the famous face test by the overall sample and as a function of age

	Age range (years)	*N* (no. female)	Mean age (*SD*)	Mean score (% correct)	*SD*	1.7-SD cut-off
Familiarity	20–29	42 (24)	25.1 (2.7)	91.89	9.64	75.50
	30–39	42 (24)	34.4 (2.9)	91.90	9.52	75.72
	40–49	42 (25)	44.2 (2.6)	94.58	6.38	83.73
	50–64	40 (25)	53.8 (3.1)	92.92	7.35	80.43
	All	166 (98)	39.2 (11.1)	92.82	8.35	78.63
Semantic	20–29	42 (24)	25.1 (2.7)	87.25	11.92	66.99
	30–39	42 (24)	34.4 (2.9)	87.23	10.82	68.84
	40–49	42 (25)	44.2 (2.6)	90.45	9.39	74.49
	50–64	40 (25)	53.8 (3.1)	89.85	9.07	74.43
	All	166 (98)	39.2 (11.1)	88.68	10.39	71.02

Split-half reliability was calculated using REL_EX_ (Steinke & Kopp, 2020), a software programme that calculates the statistic while accounting for missing data. For the purpose of reliability calculations, participants who were missing data for over 50% of the trials (i.e., reported being unfamiliar with over 50% of the famous identities) were removed from the analysis (*n* = 6). We report ρSC (the Angoff-Feldt coefficient; see Steinke & Kopp, 2020) and assume a congeneric measurement model. For familiarity judgements, the REL_EX_ analysis with 10,000 iterations revealed a median reliability estimate of ρSC = 0.819; 95% of the sampled reliability coefficients lay between ρSC = 0.718 and ρSC = 0.867. For semantic categorisation, the same analysis revealed a median reliability estimate of ρSC = 0.806; 95% of the sampled reliability coefficients lay between ρSC = 0.714 and ρSC = 0.856.

**Fig. 5:**
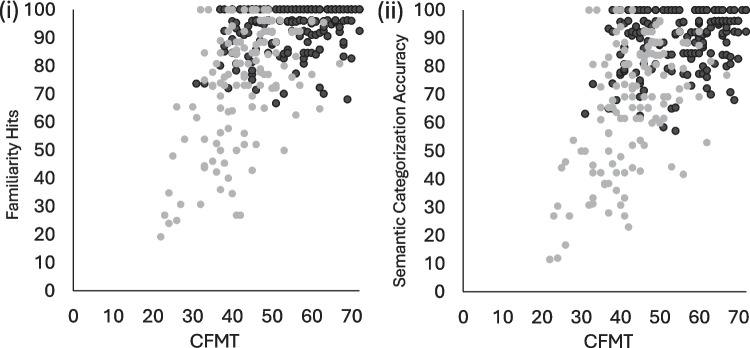
Relationship between performance in the famous face test and the Cambridge Face Memory Test (CFMT). Black circles represent typical participants (controls; Study 2). Grey circles represent self-reported DP participants (self-referred DPs; Study 3)

Convergent validity was assessed by comparing performance on the famous face test with that on the CFMT. Both the familiarity hits and semantic categorisation measures significantly correlated with performance on the CFMT (ρ = .252, *p* = .001; ρ* = *.227, *p* = .003, respectively; see Fig. 5). Divergent validity was assessed by correlating the famous face measures with the CBMT, where no significant correlations were observed (familiarity hits: ρ = .044, *p* = .574; semantic categorisation: ρ = .037, *p* = .639).

### Discussion

Study 2 aimed to develop a new version of the famous face test using the same paradigm as Study 1 and to assess its psychometric properties in individuals with typical face recognition abilities. This version of the test shows appropriate levels of performance, high reliability, and reasonable convergent and divergent validity, confirming that its psychometric properties are appropriate for use as a screening tool for prosopagnosia.

Performance across both automated measures of famous face recognition were quite high, and showed minimal decline with age (up to 64 years). These properties confirm that the task is well suited to use as a screening tool for prosopagnosia, for two reasons. First, there is substantial room between the cut-offs for impairment (78% and 71% for familiarity and semantic categorisation, respectively) and chance levels of performance for the task (50% and 8% for familiarity judgements and semantic categorisation, respectively). People with DP often report the use of compensatory strategies when learning new faces (e.g., memorising distinctive facial features; Adams et al., 2020), meaning that their face recognition is not totally abolished, but simply impaired—detecting such cases accurately is more difficult when the cut-off for classifying impairment is near chance levels of performance for a task.

Second, some measures that are commonly used to examine face processing in prosopagnosia (e.g., the CFMT) show significant age effects, such that they are not useful for diagnosis in older populations (Bowles et al., 2009). The relatively small, non-significant effects of age on this task make it suitable for screening in a broad range of individuals, without potential floor effects in some groups or the need for age-adjusted norms.

The split-half reliability for our famous face test (ρSC = 0.81–0.82) was slightly lower than reliability estimates that have been reported for the CFMT (e.g., Cronbach’s alpha = 0.87–0.89 in Bowles et al., 2009); however, they remain sufficiently high to be considered “good” for use as a clinical screening instrument (Cicchetti, 1994). Study 2 also confirmed that this version of the famous face paradigm shows good convergent and divergent validity—correlating significantly with a widely used measure of face recognition (the CFMT), but not with a measure of bicycle recognition. While the correlation between the current test and the CFMT was modest, this may reflect different properties of the tests (e.g., the use of less constrained images in this test compared to the CFMT) and the fact that they tap different aspects of face memory—encoding and short-term retrieval in the CFMT versus long-term retrieval and identification in the famous face test. Alternatively, the modest correlations might be a result of distribution of performance in the famous face test—control participants showed high accuracy and a strong negative skew, which is beneficial for detecting abnormal performance but can make it harder to discriminate between individuals within the typical range of performance.

In sum, this version of the famous face test shows good psychometric properties in a group of individuals with typical face recognition ability. Our final step was to examine the sensitivity of the task in our real-world DP screening programme, where we routinely carry out online screening of adults who report to our lab in the belief that they may experience prosopagnosia. Importantly, these individuals did not yet have a diagnosis of DP and declared that they had not participated in previous screening programmes. This ensured that they were not biased in their performance on the task by any prior experiences or information about their face recognition ability.

## Study 3

Our final study used the version of the paradigm that was developed in Study 2 and administered the task alongside other tests of face recognition ability (see below) to a group of individuals who had self-referred to our lab for an online DP assessment. To determine impairment on the task, we applied the norms that had been collected from the large group of typical participants in Study 2. This enabled us to explore the sensitivity of the famous face test in detecting face recognition difficulties in a group of individuals who believed they may experience prosopagnosia, but had not yet had this confirmed.

### Method

During the period of study, a total of 152 individuals (113 female, 36 male, 3 other) completed the online screening programme; all were UK nationals aged 20–60 years (*M* = 43.9 years, *SD* = 9.9). All participants provided informed consent to take part in the study, and ethical approval was granted by the institutional ethics committee. Participants took part in the version of the famous face paradigm that was used in Study 2 and the CFMT. Given the similarity in demographical background to the large sample of typical participants tested in Study 2, we were able to use this large dataset to establish reliable cut-offs for impaired performance in the current study. No new control participants were therefore tested in Study 3.

We were aware of one issue with the prospective DP sample that might influence the outcome of the analyses performed in this study. While most individuals who take part in our online screening programme genuinely self-refer to us in the belief that they have face recognition difficulties, we are aware that occasionally the task URLs are shared with family members who are interested in participating in the tasks but do not necessarily believe they struggle with the skill, and that other members of the general public register for screening out of general interest. This issue raised the possibility that some participants in the sample may not believe they have face recognition difficulties. We debated whether to attempt to exclude these participants, given that their inclusion might skew the outcome of sensitivity analyses. To address the issue, we used additional subjective data that we held for each participant to determine their inclusion in the final sample. That is, all participants had also completed the prosopagnosia symptom checklist (PSC: Bate et al., 2025; Murray et al., 2018), a 16-item empirically derived self-report questionnaire that is used as a brief screen for face recognition difficulties. This tool requires participants to rate how often they experience different scenarios (e.g., confusing characters in films, TV shows, or plays; avoiding using people’s names in case they have misidentified them) on a scale of 1 (never) to 5 (frequently). PSC scores were used to determine inclusion in the final sample for Study 3, with 133 participants (*M*_age_ = 43.8, *SD* = 10.2, range 20–60; 106 female, 25 male, 2 other) scoring below threshold on the PSC. Performance on the PSC was not used for any further analyses in the study.

### Results

A correlation matrix presents performance on the testing battery for the final DP sample in Table 3. We then examined performance on the famous face test using ROC analyses. These indicated that both the hits and semantic categorisation measures discriminated very well between self-reported DP and control participants (familiarity hits: AUC = 0.81, 95% CI [0.76–0.86], *p* < .001; semantic categorisation: AUC = 0.82, 95% CI [0.78–0.87], *p* < .001). Application of the 1.7-SD cut-offs from the overall control sample in Study 2 resulted in 62 participants with an impaired score on both the familiarity hits and semantic categorisation measures, eight on just the familiarity hits measure and eight on just the semantic categorisation measure. This resulted in a categorisation sensitivity *d′* of 1.44 and 1.48 respectively (out of a maximum possible *d′* of 6.10 for this dataset; see Fig. 5 and Table 4). Of the 133 participants, 55 were not impaired on either famous face measure.

**Table 3:** Correlations between task performance for the DP participants

	Famous familiarity	Famous semantic
CFMT	.487*	.468*
Famous familiarity	-	.928*

*p < .001

**Table 4:** Sensitivity of the famous face test and CFMT in detecting face recognition difficulties

		Control mean	Control *SD*	1.7-SD cut-off	DP hits	Control hits	*d′*
Famous	Familiarity	92.82	8.35	78.63	70/133	152/166	1.44
	Semantic	88.68	10.39	71.02	70/133	153/166	1.48
CFMT	1.7-SD cut-off	55.27	10.00	38	38/133	159/166	1.16
	42 raw score cut-off				62/133	143/166	1.00

When data from the CFMT were examined, ROC analyses indicated similarly good discrimination as the famous faces test (AUC = 0.81, 95% CI [0.76–0.86], *p* < .001); DeLong’s test to compare AUC was not significant when comparing between CFMT and either famous face measure, *p*s > .6. Using a 1.7-SD cut-off, 31 participants were impaired on the CFMT and both measures of the famous face test, with an additional participant impaired on the CFMT but only the familiarity famous faces measure. Six participants were impaired on the CFMT but neither famous measure. In this sample, the categorisation sensitivity *d′* of the CFMT is 1.16 (see Table 4), somewhat lower than for either of the famous face measures. Of those that performed normally on the CFMT, the famous face test identified a further 31 impaired individuals on both the familiarity hits and semantic categorisation measures, an additional seven participants only on the familiarity measure, and an extra eight only on the semantic categorisation measure (a total of 46 additional individuals). There were 49 participants who performed within the typical range on both the CFMT and famous face tasks.

Twenty-four of the control participants achieved at least one score within the impaired range (see Table 4). One participant was impaired on both famous face measures and the CFMT, six on both famous face measures but not the CFMT, one on the familiarity measure and the CFMT, one on the semantic measure and the CFMT, six only on the familiarity measure, four only on the semantic measure, and four only on the CFMT.

It should be noted that our cut-off for the CFMT, based on the control sample from Study 2, is much lower than in some previous work, and particularly those that were collected in laboratory settings. If the cut-off were allowed to be 42 (i.e., that used by many researchers in line with the norming data provided by Duchaine & Nakayama, 2006), an additional 24 individuals would be detected via the CFMT (total 62; see Table 4), 10 of whom were already identified by both the famous measures, one only by the familiarity measure, and two only by the semantic measure. Eleven of these additional participants identified by the higher CFMT cut-off were not detected by either famous measure. Thus, even with a cut-off of 42, the famous face test picked up an additional 33 individuals who were not identified by the CFMT: 21 who were impaired on both famous measures, six by only the familiarity measure, and six only by the semantic measure. With this cut-off, 38 participants performed within the typical range on both tasks. A breakdown of sensitivity and specificity for each measure and test at different cut-off scores is presented in the Supplementary Material (https://osf.io/wa56h). Notably, the optimal cut-off (calculated using Youden’s index, which balances both sensitivity and specificity of the test) varies substantially across the tasks: in our sample, the optimal CFMT cut-off is less than 0.5 SDs below the control mean, while the optimal cut-off for familiarity hits is close to 1 SD below the mean, and for semantic categorisation the optimal cut-off is around 1.5 SDs below the mean.

### Discussion

Study 3 confirmed that the novel version of the famous face test was sensitive to unconfirmed self-referred face recognition deficits—in fact, when employing the widely used cut-off of 1.7 SDs below the mean, sensitivity to self-reported deficits was higher with this task (regardless of which measure was analysed) than with the CFMT. Crucially, there was very little difference in the specificity of the task (i.e., correct classification of controls as unimpaired) between the two tasks. This was the case when using the CFMT norms based on the current control sample, and when using the commonly used CFMT cut-off of 42. Study 3 focused solely on the automatically scored measures from the task (familiarity judgements and semantic categorisation), confirming that these measures are suitable for use when screening for face recognition impairments.

Out of a sample of individuals who referred themselves to our lab and reported face recognition difficulties in the PSC, 59% also demonstrated substantial impairments on at least one measure of the famous face test. Compared to the CFMT, our famous face task identified between 33 and 39 additional participants (24.8–29.3% of the self-reported DP group) as impaired, depending on the measure and CFMT cut-off used. By comparison, the CFMT only identified between six and 11 additional participants (4.5–8.3% of the self-reported DP group) as impaired, once famous face performance was accounted for. The difference between tasks may be because the failures of face recognition that cause people to self-refer as DPs (and, to some extent, the questions used on self-report measures of face recognition such as the PSC) are more similar to those being tested in the famous face test. In short, the famous faces task may benefit from higher face validity for detecting prosopagnosia than face-learning measures.

It is also possible that familiar face recognition is a more sensitive measure for detecting difficulties with face recognition than unfamiliar face learning because of the psychometrics of the tasks: the additional cases identified by the famous faces task may represent the “milder” cases, which are easier to detect in a task with a strong negative skew such as the current famous face task (see Fig. 4). This explanation is partially supported by the observation that over half of the additional participants who were classified as impaired when the CFMT cut-off was increased had already been classified as impaired by the famous faces task. Alternatively, the additional impairments identified by the famous faces task may represent a separate subgroup of DPs—those who display specific difficulties with long-term face memory (Bate et al., 2019). Further work examining the relationship between face learning and familiar face recognition deficits would be needed to disentangle these possibilities. One of the advantages of the famous faces task presented here is that it would be comparatively simple to develop multiple parallel versions of the task with similar levels of difficulty (as in Study 1), which would facilitate more in-depth study of the relationship between different aspects of face recognition in DP, and the stability of deficits being observed.

Neither the famous face test nor the CFMT aligned perfectly with the self-report data: between 38 and 49 participants (28.5–36.8% of the self-reported DP group) did not show impairment on either behavioural task. This reflects the fact that insight into face recognition ability is not perfect (e.g., Palermo et al., 2017), and it is likely that some of the people who self-reported to our lab and scored above the cut-off on the PSC do not meet more general criteria for prosopagnosia. As such, the lower levels of sensitivity and specificity for the famous faces task in Study 2 and Study 3 likely reflect this more heterogeneous sample. We also acknowledge that our use of the PSC to determine our DP sample may also have inadvertently removed some individuals who would have met diagnostic criteria for the condition on objective tests. However, we felt it necessary to apply this step to determine our sample given that some individuals were not suspected to believe they experienced face recognition difficulties. Nonetheless, correlations between the CFMT and famous face measures were substantially higher in Study 3 than in Study 2, suggesting that the lower correlations in the control group were likely driven by the distribution of performance in that participant group, and supporting previous work advocating that relationships between tasks should be examined separately for control and DP groups (Palermo et al., 2017) .

Overall, Study 3 demonstrates that the novel version of the famous face test developed in Study 1 is also sensitive to self-reported previously unconfirmed face recognition deficits. Both familiarity judgements and semantic categorisation showed similar psychometric properties and sensitivity, suggesting that these automatically scored measures are an appropriate and effective approach for assessing familiar face recognition abilities.

## General discussion

In three studies, we validated a new famous face paradigm that can be used to automatically generate performance measures in large-scale online screening programmes. The sensitivity of the paradigm remained high across novel versions of the task, suggesting that the design can be implemented with different sets of faces according to nationality requirements. Importantly, our findings demonstrate that an automated famous face recognition test can be used in large-scale screening programmes, without the need for manual scoring or burdensome administration.

While both the familiarity judgement and semantic categorisation measures show good sensitivity and psychometric properties, there are several reasons that researchers may choose to include both measures and/or focus primarily on semantic categorisation. First, access to information about visual similarity and access to semantic information are theoretically distinct steps in the face identification process (see, e.g., the classic Bruce & Young, 1986, model of face recognition). Given that some individuals may present with more specific patterns of atypicality (Bate et al., 2019; Bennetts et al., 2022), examining both may be of particular interest for isolated cases where localisation might help to identify different subtypes of prosopagnosia/amnesia and even guide intervention (Bate & Bennetts, 2014; Brunsdon et al., 2006; Schmalzl et al., 2008). Further, the task can be readily adapted to capture free-type identification responses (e.g., names) where these data are specifically of interest for isolated case assessments.

Second, on a more practical level, the semantic categorisation task requires an additional level of engagement and person knowledge from participants, which increases confidence that good or poor performance is not simply a reflection of lucky guesses/image-based cues to fame, mistaken/rushed button pressing, or biased responding. While our analyses do not suggest a meaningful advantage of incorporating correct rejections of unfamiliar faces into the classification process (either alone or in signal detection-based measures), this leaves open the possibility that some differences between DP and control participants may be driven by a response bias (e.g., a tendency to say faces are familiar). This limitation could be dealt with by incorporating confidence or familiarity ratings into the familiarity judgement stage (which would open up the possibility of more sensitive ROC analyses on individual results). However, many researchers may prefer the simpler option of concentrating on sematic categorisation, where bias is not a significant factor. Finally, the “floor” of performance that can be meaningfully interpreted (i.e., chance performance relative to control mean performance) is substantially lower in the semantic categorisation task than in the familiarity judgement task (3.4 SDs below the control mean for familiarity; around 6.3 SDs below the control mean for semantic categorisations). As such, the semantic measure may be more appropriate for researchers who wish to examine the severity of impairment within individuals with DP (Bate et al., 2019).

Pertinently, regardless of the measure being used, the famous face recognition paradigm presented here outperformed the CFMT when it came to identifying self-referred cases of face recognition difficulties at almost every cut-off that was examined. Of course, most researchers agree that the ideal approach to diagnosing face recognition deficits is to use a combination of tasks (although opinions vary on the number and type of tasks and the appropriate cut-offs to use, e.g., Barton & Corrow, 2016; Bate et al., 2019; Burns, 2024; DeGutis et al., 2023; Lowes et al., 2024; Murray & Bate, 2020; also see Supplementary Material for optimal cut-offs for different tasks: https://osf.io/wa56h), and we do not disagree with this approach, or with the utility of using tasks such as the CFMT for rapid assessments of face recognition ability. However, we argue that screening programmes should contain a measure of familiar face recognition in order to provide a complete assessment of individuals’ face recognition skills. A familiar face recognition task may provide a sensitive index of everyday face recognition skills and escape at least some of the disadvantages associated with subjective self-report questionnaires and less ecologically valid unfamiliar face recognition paradigms, such as the CFMT. Importantly, difficulties with familiar face recognition can be extremely distressing (e.g., Adams et al., 2020; Yardley et al., 2008), with these experiences typically prompting individuals to seek diagnosis for DP. It is therefore important that assessment measures tap real-world face recognition scenarios and do not inadvertently move diagnostic criteria to reflect only a subset of the challenges that the face recognition system deals with on an everyday basis.

One crucial difference between the CFMT and our famous faces task is the distribution of performance. Data from large samples of individuals (e.g., Bowles et al., 2009; Wilmer et al., 2012) often reveal a fairly normal distribution of scores on the CFMT (although see DeGutis et al., 2023, for an exception), with the task returning high levels of reliability (e.g., Cho et al., 2015; Herzmann et al., 2008; Wilmer et al., 2012)—these characteristics make the CFMT well suited to examining individual differences in the general population. However, famous faces tasks, including the current task, tend to show a substantial negative skew (Bate et al., 2019; DeGutis et al., 2023)—control participants are generally very good at recognising and identifying famous faces, and their performance clusters near ceiling. As such, we suggest that poor performance on the current famous faces task is a useful indicator of potential face recognition impairments, and might also be useful to quantify the severity of those deficits; however, the task in its current format is not well suited to studying individual differences in the typical population, or for discriminating between those with typical and high face recognition abilities.

In sum, this investigation presents a novel, online, automatically scored face recognition test that is demonstrably sensitive for DP diagnosis and large-scale screening. We argue that familiar face recognition tests such as these should remain a core part of DP screening for theoretical and diagnostic reasons, with the present task proffering significant advantages over those previously available.

## Footnotes

1 https://yougov.co.uk/ratings/entertainment/fame/people/all. As the survey is run annually, the current list on the webpage does not exactly match the celebrities used in this study. A list of the targets can be found in the Supplementary Material (https://osf.io/wa56h).

2 There has been substantial debate about the appropriate cut-off to define impairment in face processing (e.g., Burns, 2024; DeGutis et al., 2023). In this instance we have selected conservative criteria for impairment, as we wish to examine how well the task identifies severe difficulties with face recognition. However, in light of the literature suggesting problems with overly conservative cut-offs, we have elected for a cut-off of 1.7 SDs as opposed to 2 SDs below the control mean.

3 Note that DP114 was excluded from this analysis as A and b cannot be calculated when the hit rate is lower than the false alarm rate (which is the case for this participant in Block A).

## Declarations

### Open practices statement

The data for the experiment are available at https://osf.io/wa56h. None of the experiments were preregistered.
